# Simple Preparation of a Waterborne Polyurethane Crosslinked Hydrogel Adhesive With Satisfactory Mechanical Properties and Adhesion Properties

**DOI:** 10.3389/fchem.2022.855352

**Published:** 2022-03-02

**Authors:** Jiahao Shen, Heng Zhang, Jingxin Zhu, Yanlong Ma, Hongwei He, Fengbo Zhu, Lan Jia, Qiang Zheng

**Affiliations:** ^1^ College of Material Science and Engineering, Taiyuan University of Technology, Taiyuan, China; ^2^ Shanxi-Zheda Institute of Advanced Materials and Chemical Engineering, Taiyuan, China; ^3^ Department of Polymer Science and Engineering, Zhejiang University, Hangzhou, China

**Keywords:** waterborne polyurethane, crosslinked, hydrogel adhesive, stretchability, repeatable compressibility

## Abstract

Waterborne polyurethane has been proven to be an ideal additive for the preparation of hydrogels with excellent mechanical properties. This work reports that a satisfactory adhesion of acrylamide hydrogels can be obtained by introducing a large amount of waterborne polyurethane into system. A series of polyurethane hydrogels was prepared by using one-pot method with acrylamide monomer and 2-hydroxymethyl methacrylate end-modified waterborne polyurethane emulsion. The hydrogels exhibit good strength (greater than 30 KPa), wide range of adjustable strain (200%–800%), and excellent compression fatigue resistance. The performance improvement is attributed to the fact that the polyurethane emulsion containing double bonds provides chemical crosslinking and forms polyurethane microregions due to hydrophilic and hydrophobic interactions. The hydrogel shows extensive and repeatable adhesion on diverse substrates. This simple preparation method through polyurethane crosslinked hydrogels is expected to become a low-cost and efficient preparation strategy for hydrogel adhesives.

## Introduction

As a type of soft material, hydrogel, which is crosslinked by hydrophilic macromolecular chains via covalent bond, hydrogen bond or van der Waals force with 3D network structure (Ma et al., 2016) and contains considerable water (Gong et al., 2003), exhibits low surface friction and high hydrophilicity in addition to its soft texture. Hydrogel plays a huge role in contact lenses (Hendrickson et al., 2010), tissue engineering (Chen et al., 2021), drug delivery (Li and Mooney, 2016), wound dressings (Chen et al., 2017), and other biomedical aspects.

Hydrogel adhesive is one type of special hydrogel. Hydrogel adhesives have better development prospects at hemostatic materials (Hong et al., 2019; Liang et al., 2019; Sanandiya et al., 2019), wound closure matrices (Han et al., 2017; Chen et al., 2018; Zhang et al., 2021), cartilage repair matrices (Yuk et al., 2016; Liu et al., 2017; Han et al., 2018; Cai et al., 2021; Yan et al., 2021), and other complex fields compared with general hydrogels by adhering to organic or inorganic interfaces, especially in humid conditions with a certain degree of adhesion. Understanding the formation and structure of hydrogel adhesives is of great importance to basic research and engineering applications, such as biomedicine (Han et al., 2017; Han et al., 2018; Hong et al., 2019; Liang et al., 2019), flexible supercapacitors (Tang et al., 2015), and wearable devices (Chen et al., 2019; Hu et al., 2019; Zhang et al., 2019). At present, several methods, including casting (Akindoyo et al., 2016; Rao et al., 2018; Hu et al., 2019), thermal polymerization (Han et al., 2018; Sun and Chen, 2018), and ultraviolet (UV) curing (Liu et al., 2017; Rao et al., 2018; Hong et al., 2019), have been widely used to prepare hydrogel adhesives. However, preparing simple and effective hydrogel adhesives with superior performance and repeatable bond remains challenging.

As a synthetic hydrogel, polyurethane hydrogel has been extensively used in various medical applications due to its adjustable structure, good mechanical properties, and biocompatibility. Therefore, various medical applications based on polyurethane hydrogel have been realized, from controlled drug delivery carriers (Ding et al., 2013; Sardon et al., 2015; Polo Fonseca et al., 2018) to promising or commercialized blood contact equipment materials, such as artificial heart valves, which have attracted extensive attention in recent years (Xiao et al., 2019).

Polyurethane hydrogel or hydrogel derivatives containing polyurethane, including the physical gel system of polyvinyl alcohol composite and chemical polymerization method, which inspired from incorporating acrylic double bonds to waterborne polyurethane (WPU), participating in polymerization through UV curing or free radical chain reaction, have been successfully applied in high strength and toughness hydrogel-based soft materials and other functional applications. Such mechanical properties have been raised to relatively high level in the past research. However, the adhesive properties of additive polyurethane were always ignored.

Li et al. reported the use of polyurethane emulsions as additives for strengthening and toughening of hydrogel adhesive materials (Li et al., 2021). However, the addition of polyurethane in large quantities has a negative effect on mechanical and adhesion properties.

In this work, the unsaturated double-bonded modified polyurethane emulsion as a crosslinking agent to increase the adhesion of hydrogel was reported for the first time. A water-based polyurethane crosslinking hydrogel adhesive with good mechanical properties and adhesion properties through a simple method was developed. The relationships between the properties of hydrogels and the types of polyurethanes with different synthetic ratios and the amount of hydrogels added were studied. Compared with N,N-methylene bisacrylamide (MBA) crosslinked hydrogels, their tensile properties, compressive properties, and fatigue resistance were improved to varying degrees, and the adhesive properties of hydrogels were positively correlated with the addition amount of waterborne polyurethane. The performance improvement was mainly attributed to the chemical bonding of polyurethane and the formation of phase separation polyurethane microregions. The gel adhesives prepared in this manner show extensive and repeatable adhesion on polar and nonpolar substrates. This method is expected to become a strategy to form an industrial system of polyurethane hydrogel adhesive with low price and excellent effect based on the mature industrial matching of polyurethane materials.

## Materials and Methods

### Materials

Isophorone diisocyanate (IPDI, 99%), poly (propylene glycol) (PPG 2000; average Mn∼2000), 1,4-butanediol (BDO, 99%), 2,2-bis (hydroxymethyl) propanoicacid (DMPA, 99%), triethylamine (TEA), 2-hydroxymethyl methacrylate (HEMA, 96%), and dibutyltin dilaurate (DBTDL, 95%) were obtained from Shanghai Adamas-Beta Chemical Reagent Co., Ltd. Other chemical reagents used in this work, including acrylamide (AM, 99%), N, N-methylene bisacrylamide (MBA, 99%), and ammonium persulfate (APS, 98.5%) were obtained from Shanghai Macklin Biochemical Co., Ltd. All reagents were utilized without further purification. Deionized water was prepared before use.

### Preparation of End-Modified Waterborne Polyurethane and EMWPUG

Preparation of EMWPU: A series of WPU emulsion was synthesized in a 500 ml three-necked round equipped flask equipped with a stirrer, thermometer, and a condenser. PTMG 2000, IPDI, and DBTDL (1%) as catalyst reacted at 90°C for 2 h. DMPA was dissolved in acetone, BDO was added and reacted for another 1 h at 80°C, and then HEA was added and reacted for 3 h at 70°C. TEA was fed dropwise to completely neutralize DMPA acidic groups in the PU chains for 30 min when the mixture was cooled down to 50°C. Cold deionized water was added quickly to the prepolymer solution at an agitation speed of 1,100 rpm for 30 min. The value of R (n (–NCO)/n (–OH)) was fixed at 1.32–1.39, and the solid content of PU emulsion was fixed at 28.5%–25.2% (Supplementary Table S1).

Fabrication of the hydrogels (EMWPUG R = 1.32, 1.25%): The hydrogels were prepared through a one-pot free radical copolymerization. Monomer AM (1.0 g) and crosslinker EMWPU (R = 1.32, 1.0 g) were first dissolved in water (4.0 g), and the remaining 1 g water was used for dissolving the initiator APS. The APS solution was added dropwise into the solution containing AM and EMWPU. The consequent solution was mixed and manually stirred for 1.5 min at room temperature. Next, the mixtures were degassed vacuum before injected into the glass molds. Subsequently, the molds were placed in an oven for thermal polymerization for 6 h at 60°C to attain the final EMWPU hydrogels. The water contents of the hydrogels were fixed at 78.7 wt% (Supplementary Table S2).

### Mechanical Tests

The tensile tests of hydrogels were conducted on a universal uniaxial testing machine (UTM4304X, Shenzhen Suns Technology Stock Co., Ltd.). The hydrogel was cut into 6 × 1 cm strips. The tensile rate was set to 50 mm min^−1^. Compression tests of hydrogels were performed on a universal uniaxial testing machine (ZQ-990B, Dongguan Zhiqu Precision Instrument Co., Ltd.). Cylindrical samples with 15 mm in height and diameter of 16 mm were tested at the compressive rate of 50 mm min^−1^. The cyclic compressive tests were conducted at a rate of 20 mm min^−1^ for 20 and 60 load–unload cycles and strain of 80% without intervals between successive cycles.

### Adhesive Tests

The adhesion test was conducted in accordance with the previously reported procedure (Matos-Pérez et al., 2012; North et al., 2017). The prepared gel sheets were made into square sheets of 18 × 18×2 mm. The upper and lower surfaces of gel were covered with the glass sheet with a size of 20 × 20 mm. After applying force for 20 s, the glass surface was sucked with a suction cup. The maximum force was obtained by using a tensile machine to clamp the suction cup and pull the glass sheet apart. The adhesion force test was repeated for five times. The adhesion strength (*σ*) of the hydrogel can be evaluated by using the following equation.

$$\sigma =F_{\max}/A$$

where *F*_max_ is the maximum force measured during the adhesion test, and *A* is the contact area.

### Other Characterizations

The Fourier transform infrared (FTIR) spectroscopy of the EMWPUG at the wavenumber range of 600–4,000 cm^−1^ with a resolution of 4 cm^−1^ was recorded on an infrared spectrometer (TENSOR 27, Bruker) at room temperature. The particle size distribution (PDI) and the average particle size of the EMWPU emulsion and contrast WPU emulsion were obtained through dynamic light scattering (DLS, Nanotrac Wave II, Microtrac) at room temperature. The morphology of freeze-dried samples was observed by using a scanning electron microscope (SEM, JEOL JSM-6700F, Japan) at the scanning voltage of 10.0 kv and sprayed with gold for 90 s. EMWPUG was immersed in distilled water to establish swelling equilibrium. Swelling degree was defined as *d*/*d*_0_, where *d*_0_ is the dimeter of gel prepared, and *d* is the diameter after equilibrium swelling (Muta et al., 2003).

## Results and Discussion

### Preparation of EMWPU and EMWPUG

**FIGURE 1:**
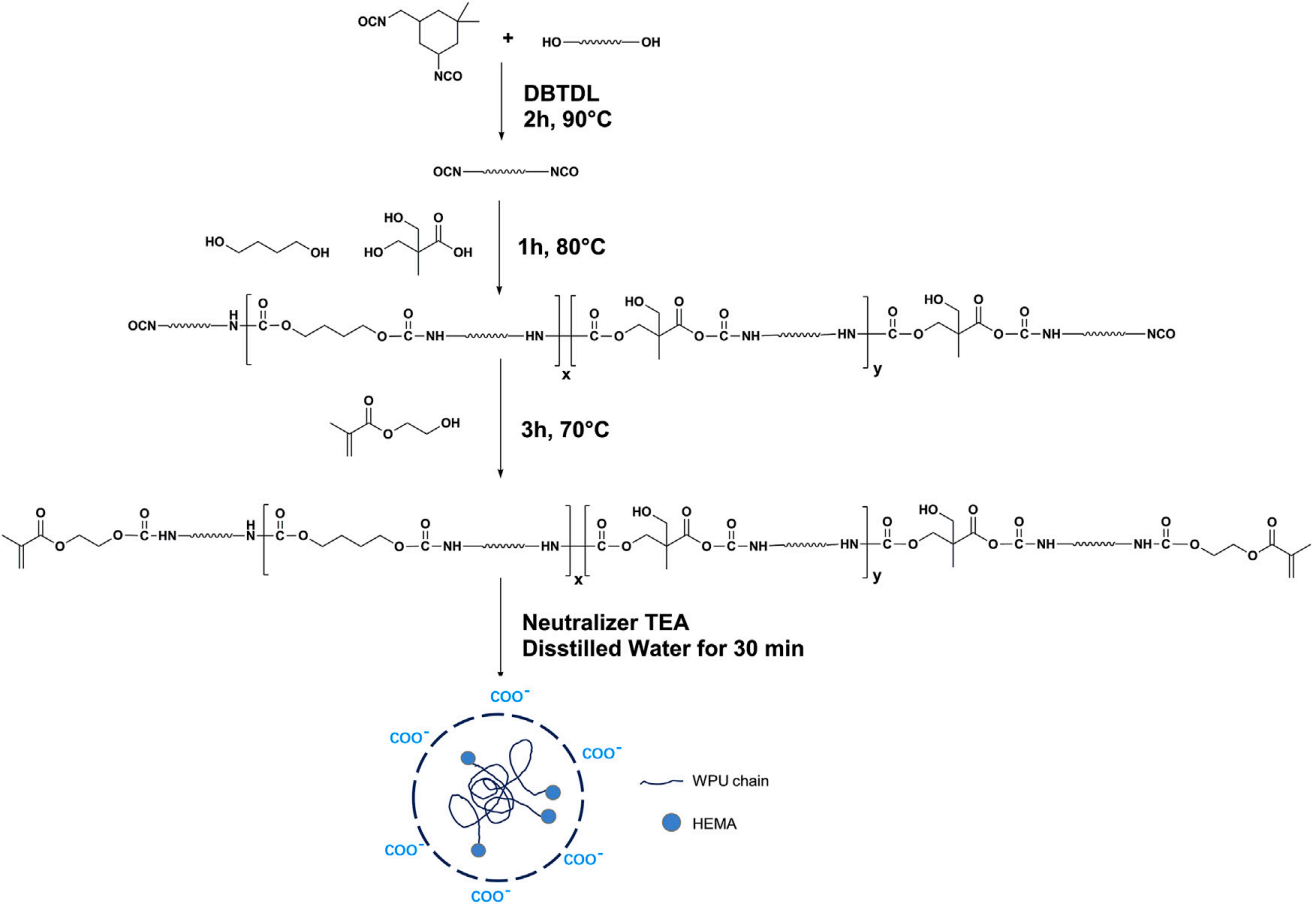
Schematic of reaction scheme for EMWPU synthesis and EMWPU emulsion microstructure.

During the synthesis process, carboxylic acid groups (–COOH) on the polymer molecular chain can be regulated by adjusting the percentage of DMPA to control the particle size of the emulsion (Sun and Chen, 2018; Dall Agnol et al., 2021). The preparation process and the structure of the emulsion are shown in Figure 1. The emulsion was prepared through prepolymerization, chain extension, end-capping, neutralization, and emulsification. After the reaction, acrylic acid became acrylate groups, changing from hydrophilic to hydrophobic. Thus, the double bonds were dispersed inside the emulsion, and the hydrophilic segment of the polyurethane chain distributed in the periphery of the emulsion.

The polyurethane chain with linear structure was prepared, and its end groups were modified with HEMA to explore the effect of polyurethane content and particle size on the mechanical properties and adhesion properties of EMWPUG. Adjusting the R value of the prepolymer can control the molecular chain length and the distribution of soft and hard segments. The isocyanate groups at two ends of the prepolymer were blocked by double bonds.

**FIGURE 2:**
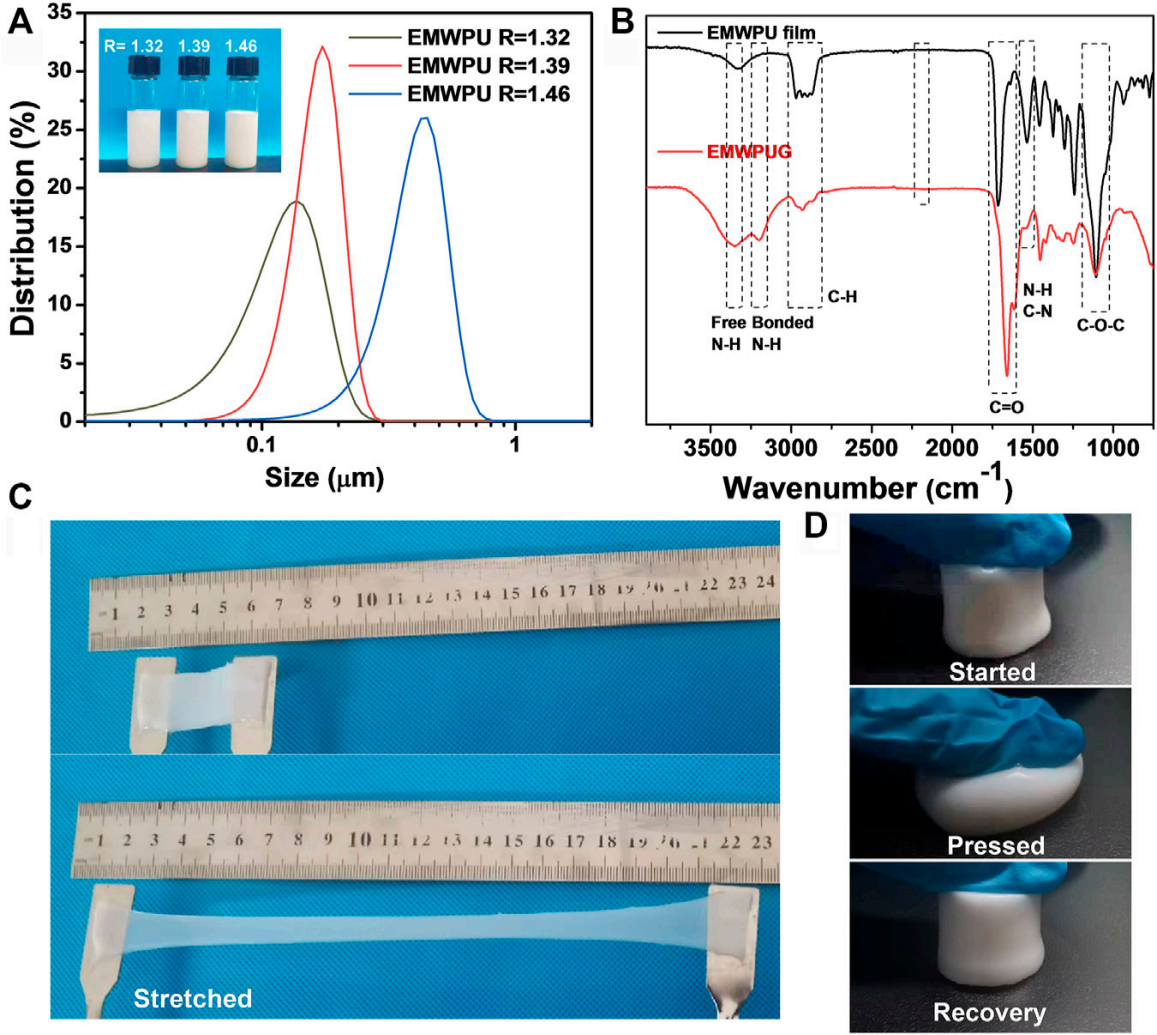
**(A)** Particle size distribution of EMWPU emulsion and samples EMWPU emulsions with different *R* values (insert). **(B)** FTIR spectrum of EMWPU film and EMWPUG after freeze-drying. Photos of the **(C)** stretchable EMWPU hydrogels and the **(D)** recovery property of EMWPUG after pressed by an index finger.

As shown in Figure 2A, the prepared blocked polyurethane emulsion was apparently milky white liquid, and the corresponding unblocked emulsion was light blue (Dall Agnol et al., 2021). And with the increase in the *R* value in the prepolymer stage, the particle sizes of the emulsion samples with *R* = 1.32, *R* = 1.39, and *R* = 1.46 were 0.126, 0.153, and 0.394 μm, respectively. The particle size of the emulsion mainly depended on the content of hydrophilic groups on the polyurethane chain. With the increase in the content of hydrophilic groups, the molecular chain was easier to stretch in water. Thus, the emulsion had a larger particle size. All the PDI values showed that the three emulsions are well dispersed. (Supplementary Table S3).

The film prepared by EMWPU emulsion and the FTIR of freeze-dried gel are shown in Figure 2B. The wide peak of 3,500–3,100 cm^−1^ was attributed to–N–H stretching vibration, and the sub peak near 3,206 cm^−1^ was attributed to hydrogen bonded–N–H stretching vibration. The strong absorption peak near 1,660 cm^−1^ belonged to the superposition of C=O and amide I bands of polyurethane and polyacrylamide; the peak at 1,537 cm^−1^ was attributed to the stretching of C–N and the bending vibration of–N–H; the peak at 1,200–900 cm^−1^ was attributed to C–O–C stretching vibration (Xiao et al., 2019). No asymmetric stretching vibration peak of NCO was found at 2,200–2,300 cm^−1^, indicating that the isocyanate group reacted completely. The split peaks appearing at 3,200 cm^−1^ and the peak position shift of C=O implied the strong hydrogen bond interactions. The information presented by FTIR showed that the polyurethane-acrylamide hydrogel was successfully prepared. The appearance of the gel is shown in Figures 2C,D. The prepared EMWPUG was homogeneous and soft, with a certain degree of toughness and can be repeatedly stretched or compressed. When the hydrogel sheet was elongated in a specific direction, a reversible color change occurs from opaque milky white to pale blue translucent. The hydrogel cylinder can quickly return to its original shape after it was compressed to the limit, thereby reflecting its toughness.

### Mechanical Properties of Hydrogel

**FIGURE 3:**
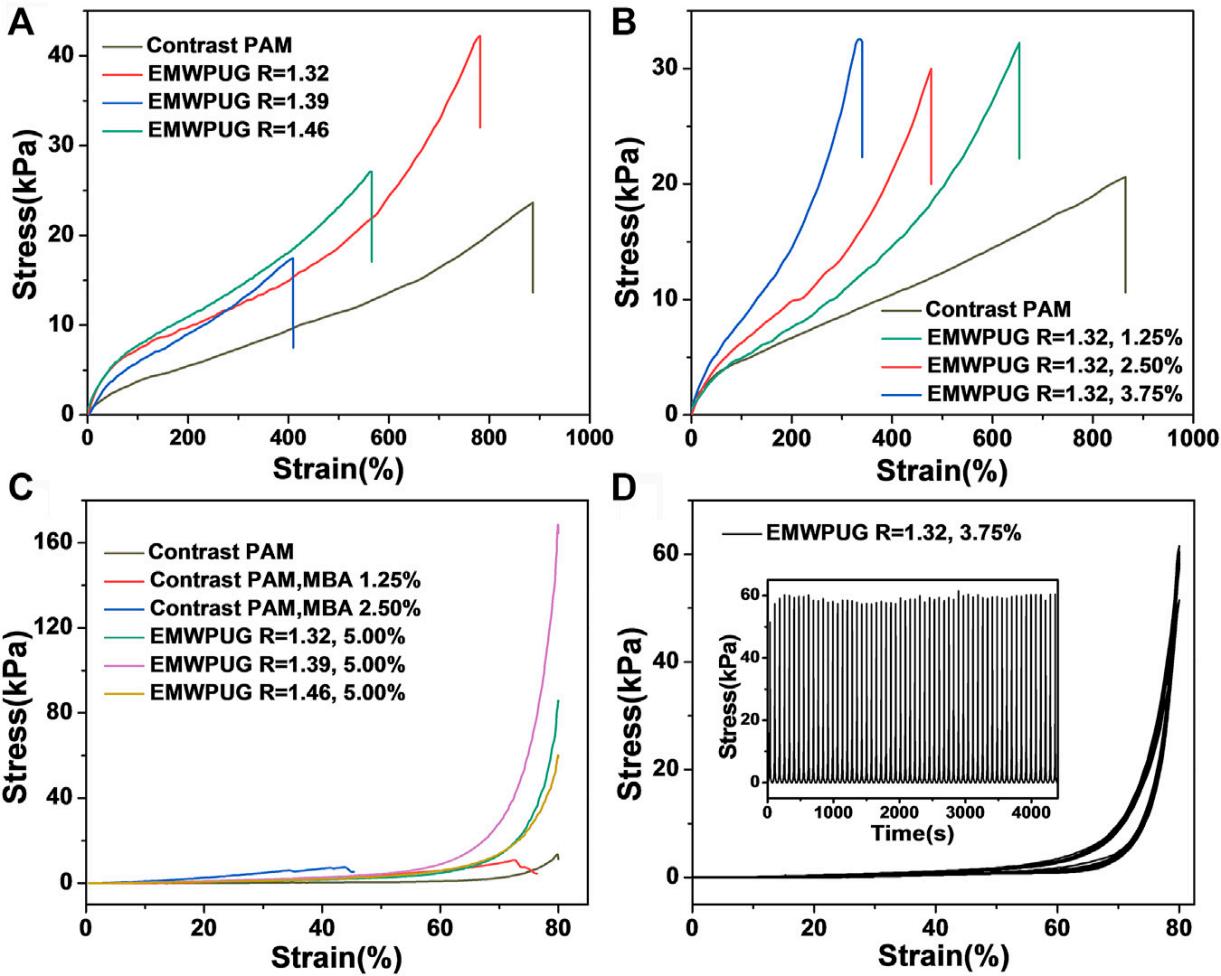
Tensile strain–stress curves of the hydrogel at **(A)** various *R* values and **(B)** various mole percentages of the double bond of EMWPU *R* = 1.32. **(C)** Compressive strain–stress curves of the EMWPUG with mole percentage of EMWPU double bond at 5.00% and the contrast samples of different mole percentages of MBA. **(D)** Compression loading–unloading test (60 cycles) of the hydrogel sample.

A series of experimental designs was developed to study the influence of the type and content of polyurethane on the mechanical properties of the hydrogel and to explore the mechanical properties of the designed hydrogel. Tensile and compression tests on EMWPUG can quantitatively characterize the mechanical properties of different hydrogels. As shown in Figure 3A, the tensile curve prepared by the gel with the same crosslinking degree shows that the tensile properties of the gel prepared by different emulsions are obviously different. The average tensile stress of the samples with *R* values of 1.32, 1.39, and 1.46 are 38.5, 19.9, and 26.1 KPa, respectively (Supplementary Figure S1).

The effect of latex particles for enhancing hydrogels has been reported previously (Gao et al., 2017; Xia et al., 2017), and the influencing factors can be summarized into two aspects: hydrophobic association and latex particle size (Liu et al., 2019). Smaller particle size of emulsion can promote the improvement of mechanical properties. Smaller particle size indicates more crosslinking sites and lower crosslinking density at each point, which will form a more uniform network to improve the mechanical properties. This condition might be the reason why *R* = 1.32 emulsion has the best enhancement of tensile properties (increased tensile strength and 700% strain at break). The tensile strengths of *R* = 1.39 and *R* = 1.46 were insignificantly different from that of the control group, and the fracture strain decreased significantly. This condition may be due to the fact that fewer crosslinking sites and the network with higher crosslinking density at the sites cannot prevent the linear acrylamide chain from being destroyed first at the low solid content.

The stretching of hydrogels of the same emulsion with different crosslinking degrees showed regular phenomena (Figure 3B). In the process of hydrogel formulation design, the ratio of AM monomer to water was kept constant while changing the solid content of blocked polyurethane. Taking EMWPUG *R* = 1.32 as an example, the difference in the slope of the curve can clearly illustrate the crosslinking effect caused by the gradual increase in emulsion, which also reduces the fracture strain significantly from 884% to 325%. The tensile stress drops from 38.5 to 27.3 KPa, which is slightly stronger than linear polyacrylamide at 22 KPa. The statistical data of multiple samples are shown in Supplementary Figure S2. When the mole fraction of double bonds involved in crosslinking was 1.25%, the sample had the best mechanical properties. The reason can be explained as below. The orientation process of the polyamide chain was hindered because the failure of these hydrogels was caused by the rupture of the linear amide chain, the ratio of acrylamide in the system remained mostly the same, the steric hindrance of the emulsion particles, and the higher degree of crosslinking. In the above experiments, as the control group, the sample containing equal mole fraction MBA crosslinking agent cannot be tested because it will be crushed directly on the fixture, illustrating the excellent performance of polyurethane as a long chain flexible crosslinking agent.

The polyurethane emulsion particles as crosslinking points can only increase the tensile performance to an acceptable level due to the single network characteristics of the hydrogel. However, in the compression test, the experimental group showed a significant improvement in compression performance compared with the control group (Figure 3C). The increase in crosslinking emulsions improves the compressive strength with different degrees. When the crosslinking percentage was 5.00%, the compressive strengths of *R* = 1.32, 1.39, and 1.46 samples were 85.3, 168.54, and 59.17 KPa, respectively, and returned to the original state after pressure unloading. For the control series of linear acrylamide gels, the compressive strengths of linear acrylamide gel, 1.25% MBA gels, and 2.5% MBA gels were 13.4, 10.9, and 7.36 KPa, respectively. The experimental group showed a high compression performance of up to 15.8 times. All the three emulsion content to compression strength characteristic curves are shown in Supplementary Figure S3. The added amount was limited to 5.00% due to the solid content limitation of polyurethane.

With the increase in the addition amount of the emulsion, the compression performance of the gel gradually increased. The mechanical properties of *R* = 1.32 group improved by tensile and compression were further studied. When the crosslinker contents were 1.25% and 2.5%, the hydrogel can recover more than 96% of the original height after 80% compression, and the experimental group with addition amount exceeding 3.75% was completely recovered. The cyclic compression characteristics of EMWPUG *R* = 1.32, 3.75% are shown in Figure 3D. The sample can withstand more than 60 cycles of 80% cyclic compression and maintain a fairly stable compressive strength, and the sample height can be fully recovered after complete pressure unloading. The 5.00% sample can go through more than 20 cycles to maintain stability and complete recovery of pressure unloading (Supplementary Figure S4). The sample with *R* = 1.39 showed a better compression performance than the average level, the gel’s compression performance was highlighted by the best dispersion, and the most uniform PDI came from the appropriate ratio of soft to hard segments. Overall, emulsion content, size, dispersibility and crosslinking density affect its compression performance. The addition of end-modified waterborne polyurethane increases not only the density of emulsion particles in hydrogel, but also the crosslinking density. The hydrogel with low content of polyurethane and crosslinking density (1.25% and 2.50%) is not enough to support itself to achieve 100% compressive deformation recovery. When the amount of polyurethane emulsion reaches 3.75%, it can achieve complete recovery and comparable fatigue resistance through mutual extrusion deformation of polyurethane emulsion particles and certain crosslinking density. When the addition amount is large (5.00%), the compression stability of the gel will decrease due to the high crosslinking density.

### Adhesive Properties of Hydrogel

Previous reports showed that the addition of polyurethane components makes the gel produce stable adhesion to the surface of the substrate. In-depth research was not conducted due to the limitation that the addition of a large number of free polyurethane components will have adverse effects on the mechanical properties of the gel. The end-modified polyurethane was proven in the previous section that it can be added to the acrylamide matrix in large quantities (up to 53% of the total solid content at most) and enhanced the mechanical properties of the main body. The influences of *R* value and content of modified polyurethane on the adhesion property of crosslinked acrylamide hydrogel as matrix and glass sheets as adhesion surface were further explored.

**FIGURE 4:**
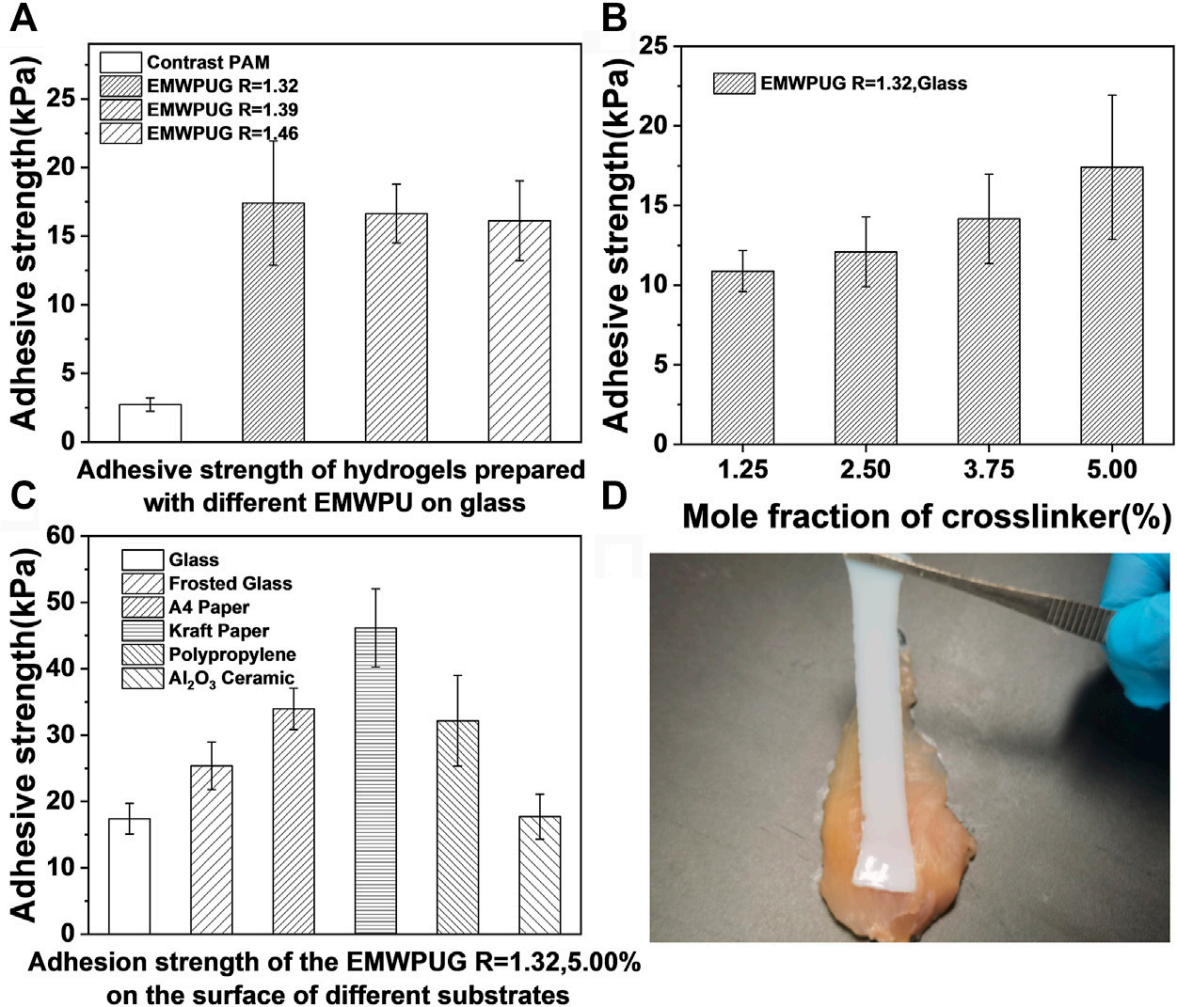
Adhesive strengths of the hydrogels with **(A)** diverse EMWPU *R* values and **(B)** different mole fractions of crosslinker (EMWPU *R* = 1.32) on glass. **(C)** Adhesive strengths of EMWPUG *R* = 1.32 at 5.00 mol fraction adhered to the surfaces of various materials. **(D)** Adhesion to chicken muscle tissue of hydrogels.

As shown in Figure 4A, the samples with *R* = 1.32, 1.39, and 1.46 all showed satisfactory adhesion, and the average values of adhesive strength were 17.4, 16.4, and 16.1 KPa, respectively. This crosslinked polyurethane had a positive effect on the bonding strength and had a wide range of adhesion to various materials. With the increase in the *R* value, the adhesion strength decreased slightly. Compared with the adhesion strength of 2.7–4.4 KPa in the control group, the adhesion strength increased by approximately one order of magnitude, proving that the adhesion was not caused by linear or netted acrylamide. A correlation was observed between the adhesion strength of the three crosslinked polyurethanes and the *R* value. With the increase in the *R* value, the adhesion strength decreased slightly. The increased *R* value led to the aggregation of adhesive groups inside the polyurethane (Yan et al., 2020) and the increase in the particle size of emulsion, which might be unconducive to its contact with the substrate interface.

The effect of polyurethane content on adhesive strength is shown in Figure 4B. The increase in crosslinking polyurethane content had a positive effect on the adhesive strength of hydrogels. Taking EMWPUG *R* = 1.32 series as the example (Supplementary Figure S5), the adhesion strength of glass increased from 10.8 to 17.4 KPa when the amount of added polyurethane increased from 1.25% to 5%, corresponding to 13%–53% of the total solid content. Hydrogels can adhere to the surface of various materials at room temperature, including organic and inorganic materials. The adhesion of different substrates was quantitatively tested. As shown in Figure 4C, the adhesion strengths of hydrogel on smooth glass, frosted glass, A4 paper, kraft paper, polypropylene, and alumina ceramics were 17.4, 25.4, 34.0, 46.2, 32.2, and 17.7 KPa, respectively. The hydrogel adhesion on the surface of animal muscle tissue is shown in Figure 4D. Observation of the hydrogel elongation proved that a significant resistance occurred during the peeling of the adhered hydrogel. The different adhesion of the gel to different materials was related to the roughness, chemical properties, and topological connection of the substrate itself (Yan et al., 2020; Yang et al., 2020; Pan et al., 2020). The universal adhesion characteristics of crosslinked polyurethane hydrogels to organic and inorganic surfaces were proven.

On this basis, the gel adhesion had repeatable ability because the gel had certain solid shape and the gel was not damaged after debonding. Taking multiple sequential content of EMWPUG *R* = 1.32, 1.25% on the glass surface as an example, the representative displacement versus adhesion curves of the hydrogels are exhibited in Supplementary Figure S6, showing three stages with growing displacement. The bonding strength increased rapidly with the increase in displacement, and then the bonding curve appeared to be flat due to the generation and development of the interface gaps, which ultimately led to the complete failure of the bonding between the interfaces. The maximum bonding force of three consecutive bonding was 3.40, 3.53, and 3.34 N, and the difference was within 6%, indicating that this material had reliable repeat bonding performance.

### Mechanism Analysis

**FIGURE 5:**
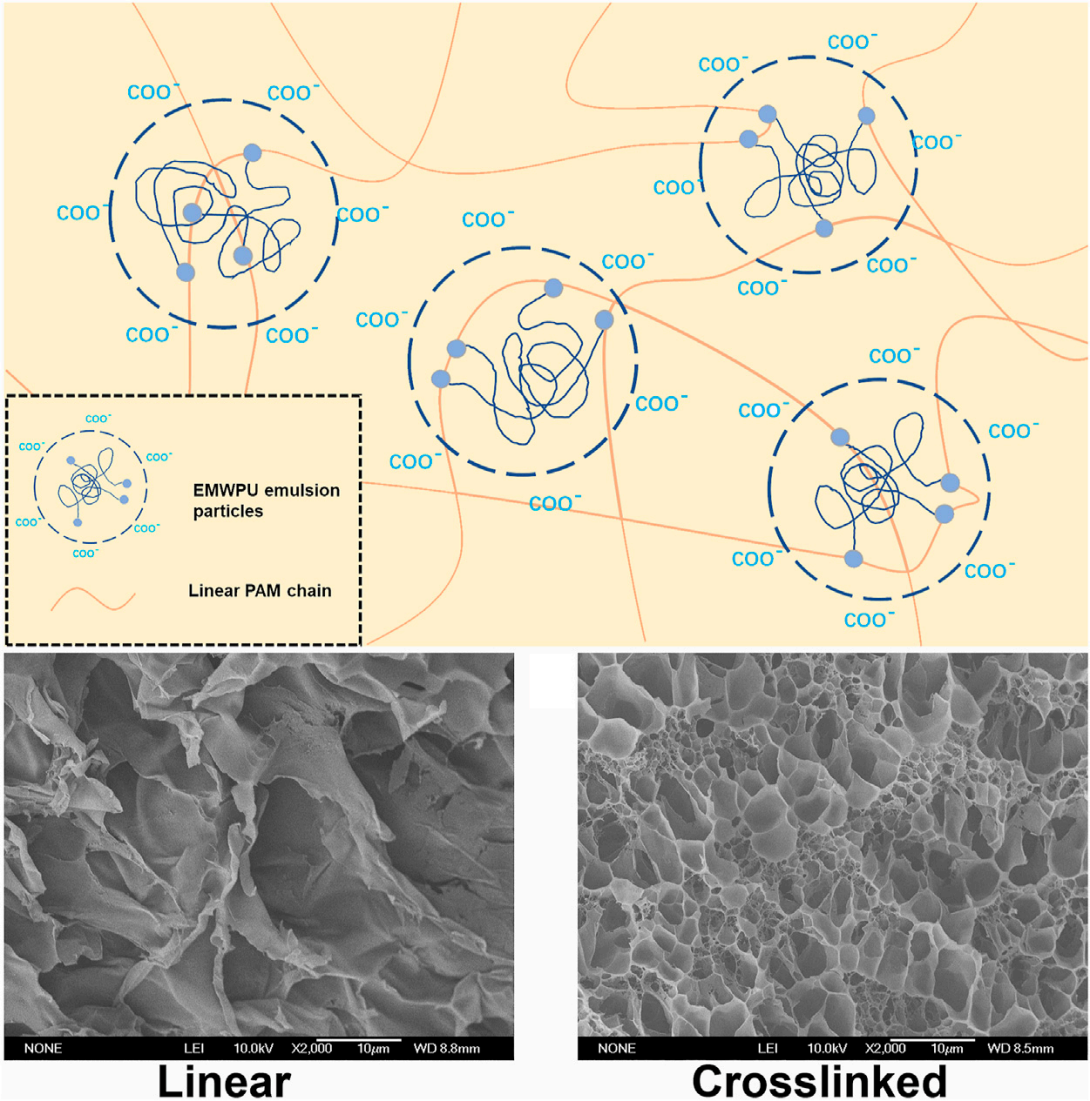
**(A)** Schematic of EMUPUG crosslinking mechanism; SEM images of **(B)** linear PAM hydrogel and **(C)** Crosslinked EMWPUG (*R* = 1.32, 1.25%).

Figure 5A shows the schematic of EMWPUG. The two ends of the curve of polyurethane (blue coil) were modified by HEMA (blue dots). The open double bond of HEMA crosslinked with linear acrylamide into the emulsion particles to form a 3D network. The waterborne polyurethane chain tended to retain its emulsion-like morphology due to its own hydrophilic and hydrophobic interactions, forming a state of microphase separation in the gel system, which can be observed in the SEM image. The SEM images of PAM (Figure 5B) and EMWPUG R = 1.32 (Figure 5C) hydrogel swelling equilibrium were enlarged by 500 times. After adding polyurethane, the crosslinking network had uniform pore size and complete pore structure, showing the crosslinking effect of polyurethane as crosslinking agent. The swelling curve (Supplementary Figure S7) showed a certain degree of antiswelling performance of polyurethane hydrogel.

**FIGURE 6:**
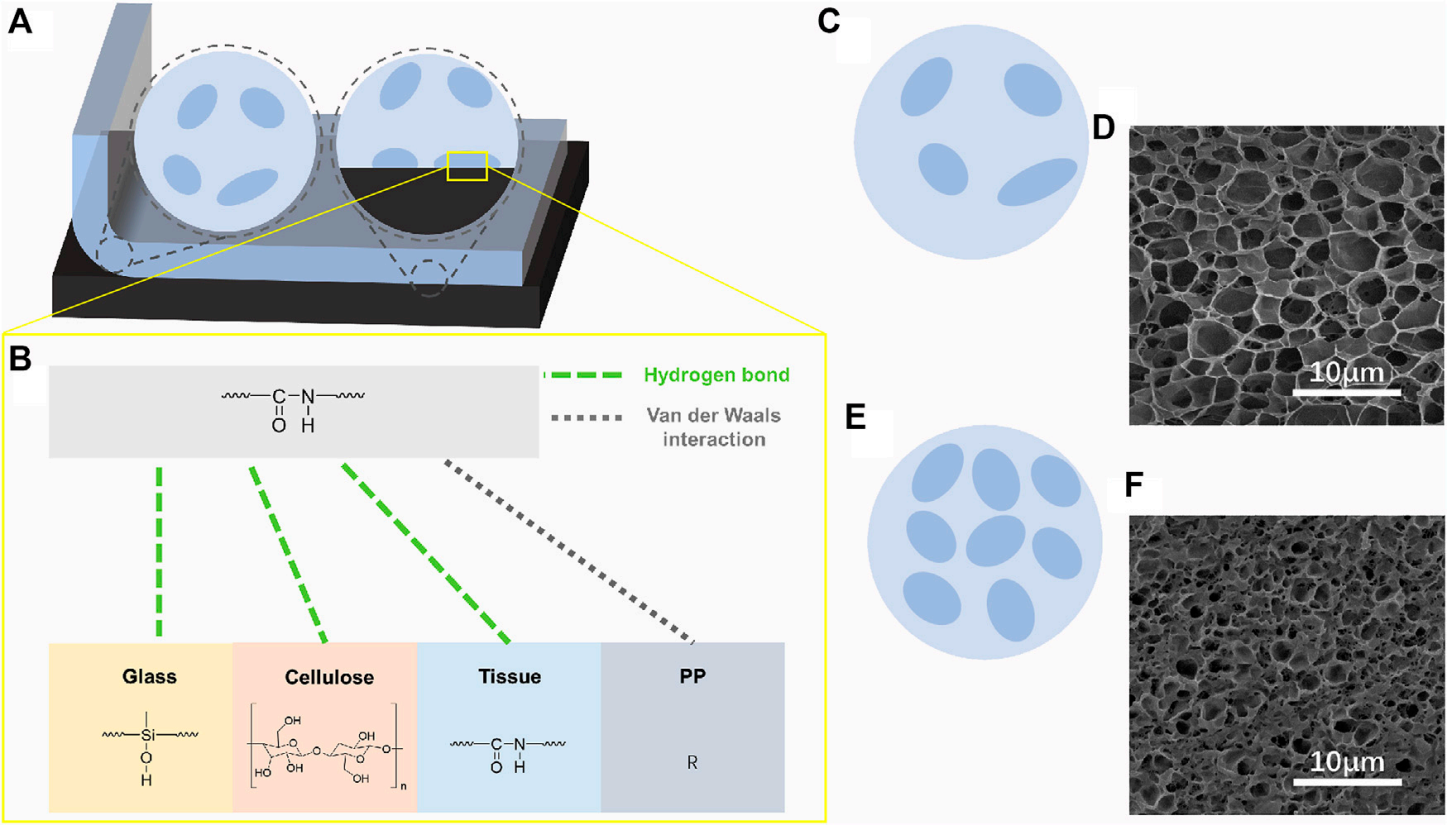
**(A)** Schematic of peeling the EMWPUG from the substrate. **(B)** Potential chemical bonds between hydrogels and different materials, including hydrogen bond and Van der Waals interaction. Schematic and pore structure of medium crosslink density hydrogel **(C,D)** and high crosslink density **(E,F)**.

As shown in Figure 6A, the hydrogel adhered to a specific matrix. Two regions, namely, the acrylamide region (light blue) and the region of individual or aggregated emulsion particles (dark blue), were found inside the gel. Phase separation resulted from hydrophilic differences between the two regions. As shown in Figure 6B, the improved adhesion of the gel relative to the acrylamide hydrogel was attributed to the polyurethane emulsion particle area exposed at the interface. Polyurethane adhered to various organic and inorganic materials through hydrogen bond or van der Waals force interaction Due to the strong polarity of the urethane group. Thus, when the content of the emulsion increased, the proportion of polyurethane on the surface of the gel increased. Therefore, the adhesion of the hydrogel increased. The existence of phase separation can be observed indirectly through SEM. After freeze-drying, the holes left by sublimation of ice template can reflect the solid content and hydrophilicity of the interval.

Figures 6C–F show the schematics of the hydrogel structure and the SEM images of the pore structure. The presence of phase separation was confirmed by the large pore region of approximately 5 μm in diameter. Figures 6C–F correspond to the middle concentration (3.75%) and the highest concentration (6.25%) polyurethane content, respectively. The small pore and macroporous microdomains belonged to relatively hydrophobic aggregated polyurethane and hydrophilic acrylamide, respectively. The small pore area was unremarkable at low solid content but was obvious at high polyurethane content and had tendency to be coherent.

The phase separation caused by the presence of these microdomains further enabled the gel to obtain good cyclic compression performance. The emulsion region formed by hydrophobic interaction had good elasticity relative to the matrix. When the network of a certain density was formed and the original linear hydrogel network was squeezed, the stress further acted on the latex balls, and the extrusion occurred between the latex balls, which was quickly recovered after the stress was removed. Crack propagation was also affected. Acrylamide microzone with low cohesion is prone to produce microcracks, and polyurethane microzone with high cohesion can prevent further propagation of microcracks (Liu et al., 2020). The microcracks generated in the linear acrylamide region can be self-healed. Thus, this hydrogel exhibited good compression fatigue resistance under the action of appropriate force.

## Conclusion

In this study, EMWPU and EMWPUG were successfully prepared by double-bonded terminated polyurethane chains. The hydrogels showed good tensile, compressive properties, fatigue resistance, and extensive repeatable adhesion to organic and inorganic substrates. Unsaturated polyurethane, as a crosslinking agent, provided a certain degree of crosslinking. The hydrophilic and hydrophobic interactions of waterborne polyurethane formed the hydrophobic polyurethane microregion in hydrogel, which has high polarity, can dissipate internal forces, and contribute to good adhesion to the interface and fatigue resistance. Avoiding the influence of complex matrix or multiple network, this work demonstrated that adding polyurethane can make hydrogels acquire the interface adhesion ability from scratch, which was not covered in previous work. Therefore, the addition of polyurethane can prepare hydrogel adhesives with good comprehensive performance, which can be used in various fields, including bioengineering. The cost can be reduced by the mature industrial matching of polyurethane and with excellent prospects.

## Data Availability

The original contributions presented in the study are included in the article/Supplementary Material, further inquiries can be directed to the corresponding authors.
