# Supplementary material for: Simple Preparation of a Waterborne Polyurethane Crosslinked Hydrogel Adhesive With Satisfactory Mechanical Properties and Adhesion Properties
Source: Front Chem. 2022 Mar 2;10:855352. doi: 10.3389/fchem.2022.855352 (PMC8924036; doi:10.3389/fchem.2022.855352)
Supplement: Supplementary file 1 [file DataSheet1.docx]

Supplementary Material

##

Supplementary Figures

## Supplementary Figure S1. The average tensile stresses of the samples with R values.

**
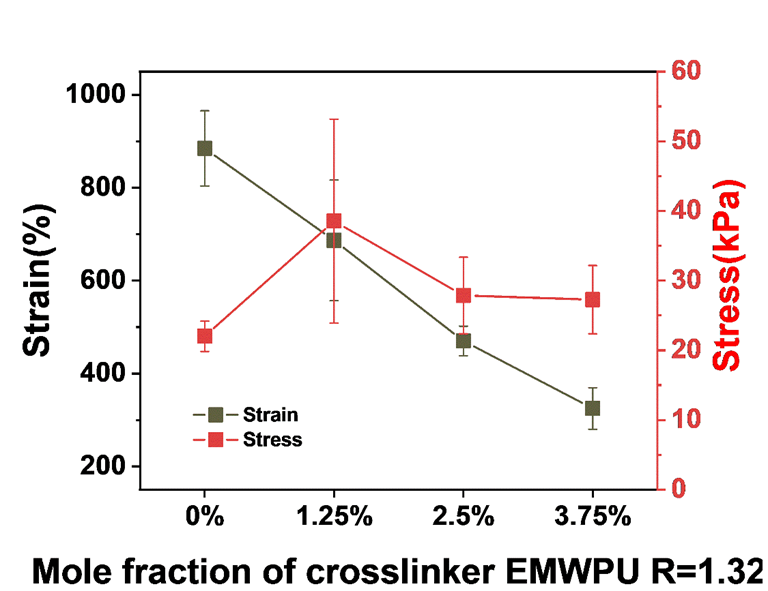
Supplementary Figure S2.** The average tensile stresses of the EMWPU *R*=1.32 samples with different percentage of double bond.


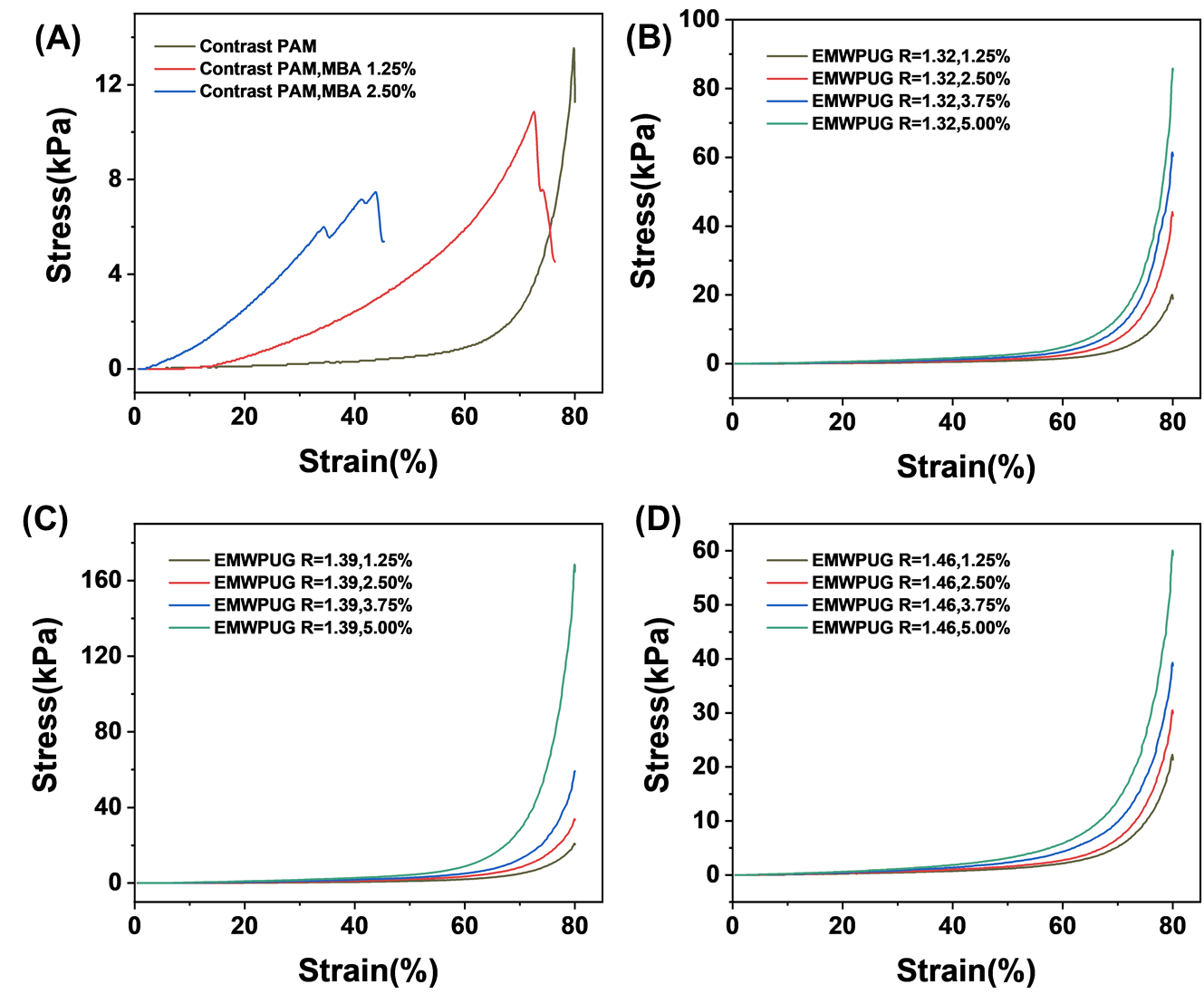


Supplementary Figure S3. Compression strength characteristic curves of (A) contrast PAM, (B) EMWPUG *R*=1.32, (C) EMWPUG *R*=1.39, (D) EMWPUG *R*=1.46 with different percentage of double bond.


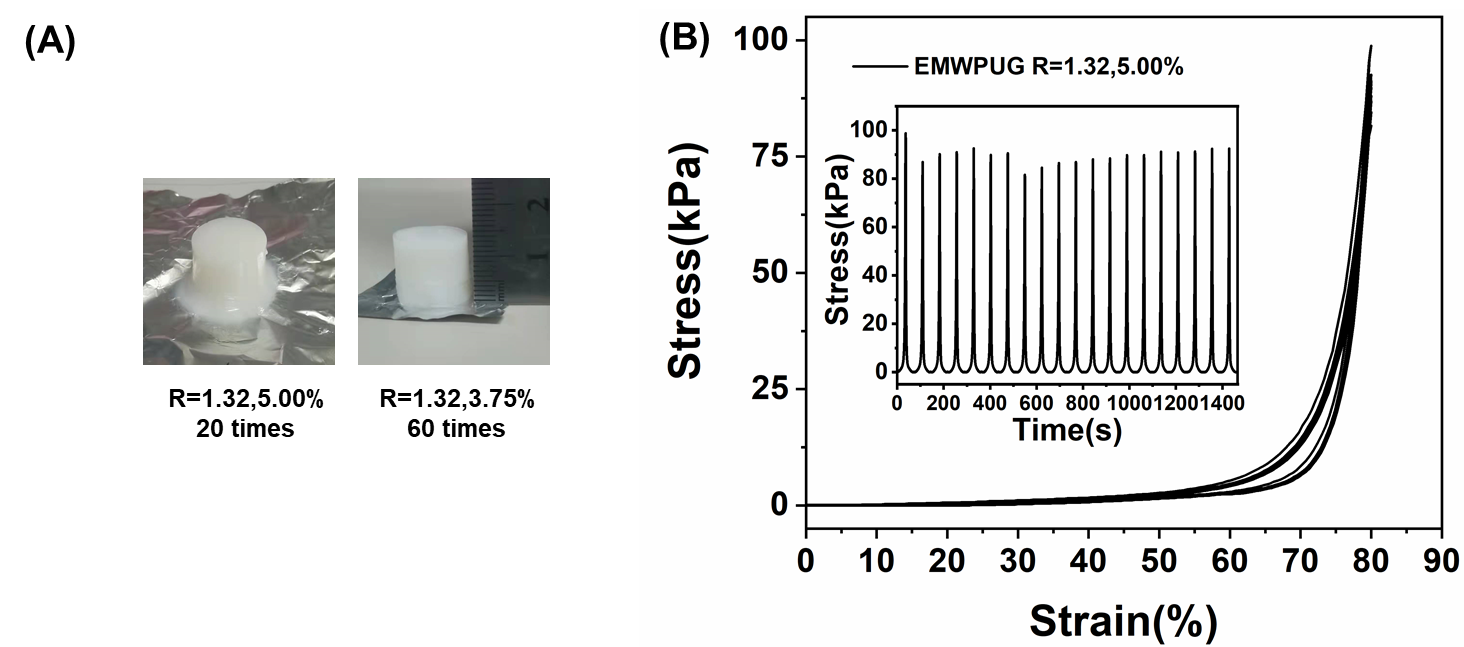


Supplementary Figure S4. (A) Samples after cyclic compression. (B) 20 times cyclic compression time-strain curve of EMWPUG *R*=1.32 with 5.00% of double bond. 60 times cyclic compression time-strain curve of EMWPUG R=1.32 with 3.75% of double bond (insert).


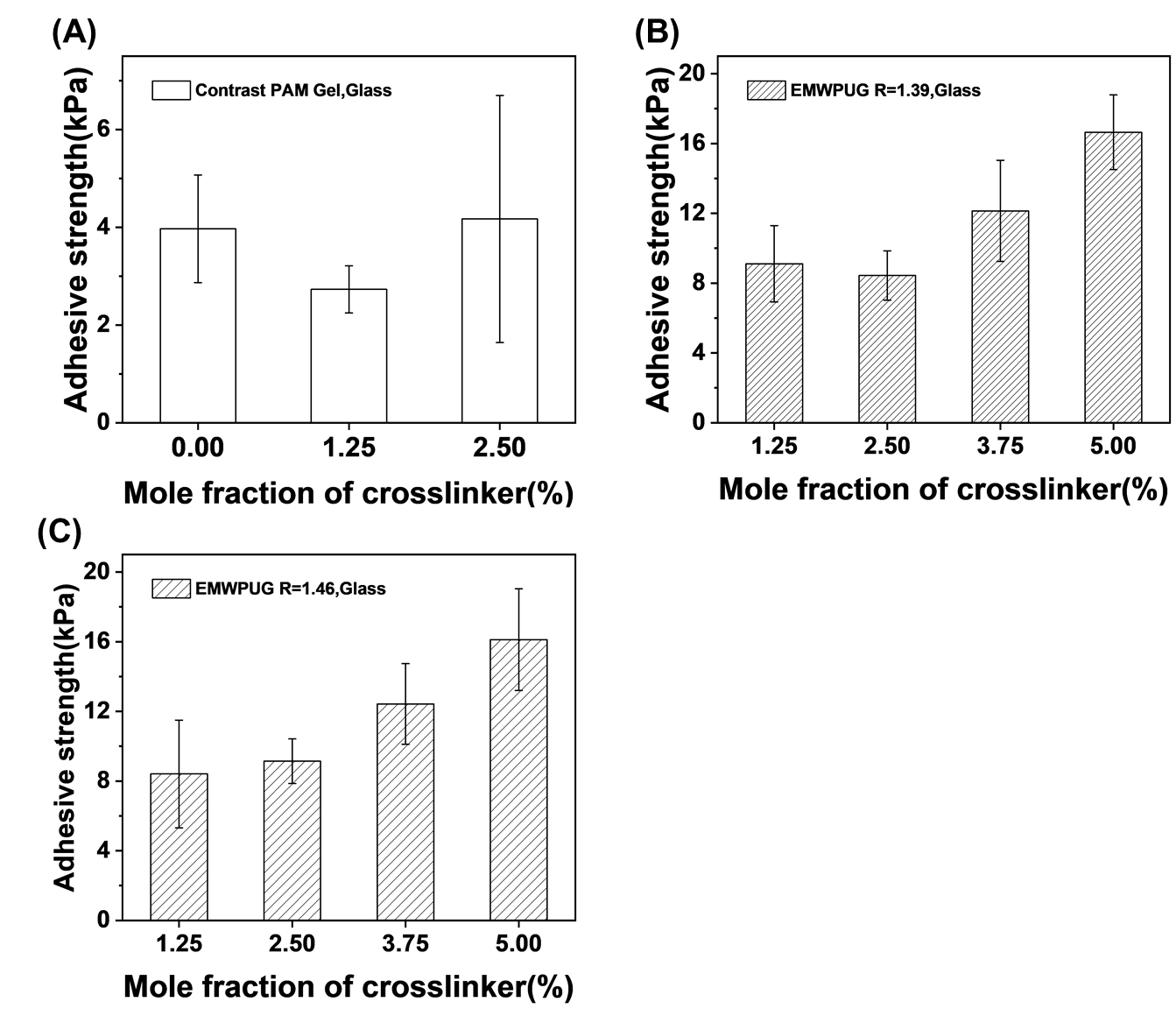


Supplementary Figure S5. Adhesion strength on glass with different *R* values. (A) contrast PAM, (B) EMWPUG R1.39, (C) EMWPUG R=1.46 with different percentage of double bond.





Supplementary Figure S6. Displacement versus adhesion force curves of EMWPUG *R*=1.32.





Supplementary Figure S7. The curve of sample swelling rate with the content of cross-linking agent.

## Supplementary Tables

Supplementary Table S1. Material parameters for preparation of emulsion samples.

| EMWPU | IPDI/g | DMPA/g | BDO/g | TEA/g | H_2_O/g | HEA/g | PPG-2000 |
| --- | --- | --- | --- | --- | --- | --- | --- |
| R=1.32 | 10.5 | 2.6 | 0.6 | 2 | 100 | 2.9 | 21.3 |
| R=1.39 | 10.5 | 2.6 | 0.6 | 2 | 100 | 3.4 | 17.8 |
| R=1.46 | 10.5 | 2.6 | 0.6 | 2 | 100 | 3.9 | 14.2 |

The value of R can be calculated by the following formula:

R = n _(–NCO)_/n _(–OH)_ = n _(IPDI)_/[n _(PPG)_+n _(DMPA)_+n _(BDO)_].

Supplementary Table S2. Material parameters for preparing hydrogel samples.

| EMWPUG | AM/g | APS/g | EMWPU R=1.32/g | | H2O/g |
| --- | --- | --- | --- | --- | --- |
| 0% | 2.5 | 0.075 | 0 | 15.00 | |
| 1.25% | 2.5 | 0.075 | 2.5 | 13.22 | |
| 2.50% | 2.5 | 0.075 | 5.0 | 11.44 | |
| 3.75% | 2.5 | 0.075 | 7.5 | 9.66 | |
| 5.00% | 2.5 | 0.075 | 10.0 | 7.88 | |

The mole fraction of double bonds (n _(C=C)_ %) in samples can be calculated by the following formula:

c _(C=C)_ =n _(HEMA)_ = (2n _(–CNO)_ -2n _(–OH)_)/M = [2n _(IPDI)_ -2(n _(PPG)_+n _(DMPA)_+n _(BDO)_)]/M

n _(C=C)_ % = 100%*m*c _(C=C)_/n _(AM)_

Where c _(C=C)_ is the concentration of double bond, n _(HEMA)_ is the amount of substance of HEMA, M is total mass of the emulsion, m is the mass of the amount of emulsion added, n _(AM)_ is the amount of substance of acrylamide.

Supplementary Table S2. PDI values of different emulsions.

| Samples | PDI value |
| --- | --- |
| EMWPU R=1.32 | 0.622 |
| EMWPU R=1.39 | 0.291 |
| EMWPU R=1.46 | 0.840 |
